# Integrative analysis links autophagy to intrauterine adhesion and establishes autophagy-related circRNA-miRNA-mRNA regulatory network

**DOI:** 10.18632/aging.204969

**Published:** 2023-08-23

**Authors:** Xiaotong Peng, Yiping Zhu, Tao Wang, Shuo Wang, Jing Sun

**Affiliations:** 1 Shanghai Key Laboratory of Maternal Fetal Medicine, Shanghai Institute of Maternal-Fetal Medicine and Gynecologic Oncology, Shanghai First Maternity and Infant Hospital, School of Medicine, Tongji University, Shanghai 200092, China; 2 Department of Gynaecology and Obstetrics, Xiangya Hospital, Central South University, Changsha 410008, China; 3 Department of Orthopedics, Shanghai Sixth People's Hospital Affiliated to Shanghai Jiao Tong University School of Medicine, Shanghai 200233, China

**Keywords:** intrauterine adhesion, autophagy, circRNA-miRNA-mRNA, regulatory network, bioinformatics

## Abstract

Background: Intrauterine adhesion (IUA) is a troublesome complication characterized with endometrial fibrosis after endometrial trauma. Increasing number of investigations focused on autophagy and non-coding RNA in the pathogenesis of uterine adhesion, but the underlying mechanism needs to be further studied.

Methods: mRNA expression profile and miRNA expression profile were obtained from Gene Expression Omnibus database. The autophagy related genes were low. Venn diagram was used to set the intersection of autophagy genes and DEGs to obtain ARDEGs. Circbank was used to select hub autophagy-related circRNAs based on ARDEMs. Then, the differentially expressed autophagy-related genes, miRNAs and circRNAs were analyzed by functional enrichment analysis, and protein-protein interaction network analysis. Finally, the expression levels of hub circRNAs and hub miRNAs were validated through RT-PCR of clinical intrauterine adhesion samples. *In vitro* experiments were investigated to explore the effect of hub ARCs on cell autophagy, myofibroblast transformation and collagen deposition.

Results: 11 autophagy-related differentially expressed genes (ARDEGs) and 41 differentially expressed miRNA (ARDEMs) compared between normal tissues and IUA were identified. Subsequently, the autophagy-related miRNA-mRNA network was constructed and hub ARDEMs were selected. Furthermore, the autophagy-related circRNA-miRNA-mRNA network was established. According to the ranking of number of regulated ARDEMs, hsa-circ-0047959, hsa-circ-0032438, hsa-circ-0047301 were regarded as the hub ARCs. In comparison of normal endometrial tissue, all three hub ARCs were upregulated in IUA tissue. All hub ARDEMs were downregulated except has-miR-320c.

Conclusions: In the current study, we firstly constructed autophagy-related circRNA-miRNA-mRNA regulatory network and identified hub ARCs and ARDEMs had not been reported in IUA.

## INTRODUCTION

Intrauterine adhesion (IUA) was mainly attributed to mechanical injury and infection-induced damage to endometrial basal layer, resulting in a series of abnormal clinical manifestations including cavity blockage, hypomenorrhea and recurrent pregnancy loss [1]. The implantation of anti-adhesion membranes can inhibit the migration of myofibroblast into the injury sites in various adhesion diseases [2]. However, foreign body reaction and chronic inflammation were appeared with long-term of biomaterials implantation [3]. Although transcervical resection of adhesions (TCRA) can effectively remove adhesion tissue, the postoperative recurrence rate is as high as 60% [4]. The optimal treatment remains an ongoing debate for avoiding the postoperative vicious cycle of “adhesion-release-readhesion”, which seriously harms the reproductive health of women during the period of duration [5]. Unfortunately, the underlying pathogenesis of IUA is still uncharacterized and there are no therapeutic targets and precision drugs available for clinical screening and individualized treatment.

Autophagy is a homeostatic process in which proteins or organelles in the cytoplasm are transported to lysosomes for degradation, and then degraded contents are recycled to meet the metabolic needs and the renewal of organelles [6]. Aberrant regulation of autophagy is involved in the progress of multiple adhesive diseases, including IUA [7], peritendinous adhesion [8] and abdominal adhesion [9]. Therefore, targeted therapy towards autophagy is in critical need for IUA. As the main pathological manifestations of IUA, excessive collagen deposition and fibrogenesis are closely related to dysfunctional autophagy in multiple types of endometrial cells [10]. Recent investigations have demonstrated that DIO2-MAPK/ERK-MTOR signaling pathway regulated the defective autophagy of endometrial epithelial cells to promote their epithelial-mesenchymal transition (EMT), leading to IUA [11]. In addition, inhibition of autophagy in endometrial stromal cells (ESCs) induced the hyperactivation of hedgehog pathway and exacerbate IUA [7]. Above findings indicated that autophagy exerted a key function in the pathological process of IUA and was regarded as the potential target for IUA treatment.

Non-coding RNAs (ncRNAs), including circRNAs and miRNAs, have been extensively studied in recent years [12]. As miRNA sponges, circRNAs could competitively bind to response elements of miRNA and then inhibit the silencing of downstream target genes by miRNAs [13]. In recent years, the role of circRNA-miRNA-mRNA regulatory network in regulating biological processes, such as transcriptional regulation, post-transcriptional regulation and protein modification, has been widely concerned in adhesion diseases. Previous study indicated that overexpression of circPTP4A2 could accelerate mitochondrial metabolism through inhibition of miR-330-5p-PDK2 pathway to reduce fibrosis area and improve glands number in IUA [14]. Moreover, circPlekha7 relieved IUA through contributing to ESCs apoptosis and inhibiting myofibroblast transdifferentiation and collagen deposition [15]. Accumulating evidence has indicated that dysregulation of miRNAs is involved in IUAs. miR-1291 silenced ArhGAP29-RhoA/ROCK1 pathway to promote EMT and then aggravate IUA [16]. In addition, Li et al. found that miR-29b downregulated TGF-β1-Smad2/3 pathway of ESCs to alleviate TGF-β1-induced IUA [17]. However, there has been few reports on the autophagy-related regulatory network between circRNAs, miRNAs and mRNA in intrauterine adhesion.

The widespread application of global transcriptome analysis helps us delve deeply into how diseases form and progress. Regulatory network analysis screens the key signaling pathway, elucidates biological function and discovers new uses of old drugs in the intricate relationship for optimizing treatment of patients and achieving clinical translation. As indicated in overall flowchart (Scheme 1), our study aimed to establish autophagy-related circRNA-miRNA-mRNA network and reveal the novel pathogenic mechanism of autophagy-related circRNAs in IUA through bioinformatics analysis, and also predicted new potential drug targets. Besides, we also aimed to validate function of these autophagy-related circRNAs through clinical samples and *in vitro* experiments. In conclusion, the purpose of this study was to bridge the gap in clinical screening and precise treatment via unscrambling autophagy-related circRNA-miRNA-mRNA network for improving the female reproductive health.

## MATERIALS AND METHODS

### Data collection

After carefully screening in Gene Expression Omnibus (GEO) database (http://www.ncbi.nlm.nih.gov/geo/) and ArrayExpress database (https://www.ebi.ac.uk/arrayexpress/), mRNA expression profile (GSE160633) and miRNA expression profile (GSE160634) were obtained. In both datasets, each sample (GSM4876242, GSM4876243, GSM4876244 and GSM4876245) contained adhesion tissues or adjacent normal tissues from 8 patients. The detailed information of two microarray datasets was shown in Table 1.

**Table 1:** Characterization of the two datasets from GEO database.

**Accession**	**Platform**	**Sample**	**Normal endometrium**	**Adhesive endometrium**	**mRNA/ microRNA**
GSE160633	GPL11154	Tissues	8	8	mRNA
GSE160634	GPL18573	Tissues	8	8	microRNA

### Screening of ARDEGs

With the threshold (absolute log_2_fold change > 1), 1093 DEGs were screened from GSE160633 in the comparison between IUA tissues and normal tissues. The selected criterion was in line with the previous study [18]. The autophagy related genes were downloaded from the Human Autophagy Database (http://www.autophagy.lu/index.html). The relevant information of autophagy genes was summarized in Supplementary Table 1 and Supplementary Figure 1. Venn diagram [19] was used to set the intersection of autophagy genes and DEGs to obtain ARDEGs. Furthermore, the heatmap of ARDEGs was visualized by SangerBox Database (http://sangerbox.com/). MapGene2Chrom (http://mg2c.iask.in/mg2c_v2.1/) is an online tool for mapping the chromosomal location of genes and was applied to map the location of ARDEGs on human chromosomes.

### Construction of the autophagy-related protein-protein interaction (PPI) network

Interaction relationships between ARDEGs were visualized by STRING database (http://www.string-db.org/), a searching tool based on confidence score for the retrieval of interacting genes/proteins. Cytoscape software vision 3.9.1 was utilized to construct the PPI network.

### Functional enrichment analysis

To further clarify the functional annotation of Autophagy-Related DEGs, Gene Ontology (GO) functional annotation and Kyoto Encyclopedia of Genes and Genomes (KEGG) pathway enrichment analysis was conducted in the Sangerbox database (http://sangerbox.com/Tool). The enriched functions of hub ARCs were mainly assessed through the enrichment analysis of downstream target genes regulated by hub ARCs. GO functional analysis was mainly classified into cellular components (CC), biological processes (BP), and molecular functions (MF).

### Drug-ARDEGs network construction

In order to explore the target drugs of ARDEGs, the drug-gene interaction database (DGIdb, https://dgidb.genome.wustl.edu/) was adopted, which is the online tool for drug-gene predication. Finally, Cytoscape was utilized to construct the drug- ARDEGs network as previously described [20].

### Homology modeling and molecular docking

Alphafold database (https://alphafold.ebi.ac.uk/) was utilized to predict protein crystal structures based on amino acid sequence [21]. Unfortunately, the crystal structures of human HIF1A and PRKCQ have not yet to be work out. Therefore, the structure of human HIF1A and PRKCQ was constructed through *in silico* modelling in Alphafold database. The amino acid sequences of human HIF1A and PRKCQ (accession numbers: Q16665 and Q04759, respectively) were obtained from the UniProtKB database (http://www.uniprot.org/). The local distance difference test (LDDT) score was adopted to evaluate the stereochemical quality of human HIF1A and PRKCQ structure. Based on drug- ARDEGs network, associated drugs were downloaded from the Pubchem database (https://pubchem.ncbi.nlm.nih.gov). Molecular docking was utilized to screen the best drugs to achieve the target therapy of HIF1A and PRKCQ. Molecular docking between potential drugs and HIF1A or PRKCQ binding pockets was performed by Autodock Vina with default parameters. The predicted binding interaction geometries of ML228 towards HIF1A and staurosporine towards PRKCQ were visualized and the docking affinity was scored. PyMol was applied to visualize the optimal drug-protein conformation as previous described [22].

### Screening of ARDEMs and construction of autophagy-related miRNA-mRNA regulatory network

With the same criterion, 113 DEMs were screened from GSE160634. miRWalk3.0 (http://zmf.umm.uni-heidelberg.de/apps/zmf/mirwalk3/) was used to predict target miRNA of ARDEGs. ARDEMs were screened by overlapping the predicted target miRNAs and DEMs. In addition, the ARDEMs-target ARDEGs regulatory networks were visualized by Cytoscape. In this regulatory network, miRNAs with the largest number of regulated ARDEGs were considered as the hub ARDEMs.

### Functional and pathway enrichment analysis of hub ARDEMs

MiRpath (https://snf-515788.vm.okeanos.grnet.gr/) is an online tool to perform enrichment analysis of miRNA. By entering hub ARDEMs ID, the visualization of GO and KEGG enrichment analysis was performed with the threshold (adjusted p < 0.05).

### Selection of hub ARCs and construction of autophagy-related circRNA-miRNA-mRNA regulatory network

Circbank (http://www.circbank.cn/index.html) is a database that integrates circRNA -related information from the entire human genome. It was used to predict ARCs based on ARDEMs. Due to the large number of ARCs, ARCs regulating more than 6 ARDEMs were included and visualized in autophagy-related circRNA-miRNA-mRNA regulatory network. Among ARCs in this network, we selected the ARCs with the highest number of regulatory ARDEMs ranking top three as the hub ARCs. At the same time, KEGG and GO enrichment analysis was performed.

### Tissue samples

IUA tissue was obtained from 5 patients who were undergoing transcervical resection of adhesions in Xiangya Hospital. Normal endometrium was obtained from healthy women of reproductive age in Xiangya Hospital. None of the patients was on hormonal medications. All the biopsy specimens (both normal and IUA) were obtained during the early proliferation phase of each woman’s cycle. All samples were taken with the consent of the patients, and informed consent was signed. Consent was granted by all women included in the study, and informed consent was signed. All procedures were approved by the Medical Ethics Committee at Xiangya Hospital, Central South University (2022111228). Before surgery, all patients underwent B-ultrasound examination. All diagnoses of IUA were confirmed by hysteroscopy. No patients received any treatment before the surgical procedure. The IUA tissue was carefully excised under the hysteroscopy by the annular electrode, and normal endometrium was carefully excised by medical curette. Tissues were stored at −80° C immediately after harvest until further analysis.

### Hematoxylin-eosin staining

After fixed for 24h, the tissues were dehydrated with gradient alcohol. The tissues were embedded using paraffin wax and subsequently cut into 5μm slices using a microtome. The slices were stained with the H&E staining kit (Beijing Sola Biotechnology Co., Ltd., Beijing, China). Five images were randomly captured under a light microscope (Leica DMi6-M, Leica Microsystems Co., Ltd.) to observe the pathological changes in IUA.

### RNA extraction and RT-PCR validation

Total RNA extraction kit (Shabio, Shanghai, China) was used to extract RNA from tissues and cells based on instructions. Total RNA was then digested with RNase R (Epicenter, Madison, WI, USA) and reversely transcribed into cDNA with NovoScript II Reverse Transcriptase (E126-01A, Novoprotein, Suzhou, China) for circRNA and mRNA, and miRNA 1st Strand cDNA Synthesis Kit (MR201-01, Vazyme Biotech, Nanjing, China) for miRNA. RT-PCR was performed on hub ARCs, hub ARDEMs and associated mRNA in an ABI PRISM® 7500 Sequence Detection System (Applied Biosystems Inc., Foster City, CA, USA) using AceQ Universal SYBR qPCR Master Mix (Vazyme Biotech, Nanjing, China) and miRNA SYBR qPCR kits (Leadlab, Beijing, China). Replicate wells were set up for each sample. The PCR reaction programs: For hub ARCs and mRNA, 60 s at 95° C, 40 cycles for 45 s at 60° C, 60 s at 72° C and a final cycle for 10 min at 72° C; For ARDEMs, 3 min at 95° C, 40 cycles for 15 s at 95° C, 40 s at 60° C. GAPDH and U6 were used as the internal normalization controls for hub circRNAs, mRNA and hub DEMs detection, respectively. 2−ΔΔct method was used to determine relative fold expression of circRNA. The primer sequences are synthesized by Sangon Biotech (Shanghai, China) and are shown in the supplemental material (Supplementary Table 2).

### Cell culture and siRNA transfection

The human endometrial stromal cells (ESCs) were obtained from Procell (Wuhan, China). The cells were cultured in DMEM medium containing 10% FBS and 1% penicillin-streptomycin inconstant temperature and humidity environment (37° C and 5% CO_2_). When cell confluence reached about 70% in 24-well plates, the cells were treated with TGF-β (5μg/mL, Sino Biological Inc., Beijing, China) 24h to simulate the microenvironment of intrauterine adhesion and transfected with lipo 3000/siRNA to knockdown the expression of hub ARCs and observe the autophagy level and myofibroblast transformation. The cells were randomly divided in to six group as follows: 1) Control; 2) TGF-β; 3) TGF-β+siRNA-NC; 4) TGF-β+siRNA-hsa-circ-0047959; 5) TGF-β+siRNA-hsa-circ-0032438; 6) TGF-β+siRNA-hsa-circ-0047301; Above siRNA was synthesized by GenePharma (Shanghai, China).

### Immunofluorescence

After fixation with a stationary liquid, permeabilization with 0.3% Triton X100, and blocking with 5% BSA solution, cultured cells were incubated overnight with the α-SMA (1:200 dilution, ABclonal, Wuhan, China) primary anti-rabbit antibodies at 4° C. After a brief wash with PBS, cells were incubated with fluorescent secondary antibodies for 1 h and then stained with DAPI (1 mg/ml, Beijing Sola Biotechnology Co., Ltd., Beijing, China) for 10 min at room temperature. All antibodies mentioned above were diluted in NCM universal antibody diluent from New Cell and Molecular Biotech (Jiangsu, China). Immunofluorescence was recorded by a fluorescence microscope. The percentage of α-SMA positive cells was calculated by Image-Pro Plus (Media Cybernetics, Rockville, MD, USA).

### Enzyme-linked immunosorbent assay

Collagen III (Col III) [23] and fibronectin 1 (FN1) [24] are the biomarkers of IUA. ELISA was performed to measure the concentrations of associated indicators (Col III, and FN1, ABclonal Biological Technology Co., Ltd, Hubei, China; Phospho-Beclin 1, total Beclin 1, p62 and LC3B, Abcam, USA) in supernatant. Briefly, individual antibody-coated microtiter plates were immersed with each diluted sample (100μl) and then placed in a 37° C thermostat. After a 90min incubation and four times washing, 100 μl/well biotinylated working solution was added to the plates, which were sealed and incubated for 1h in a thermostat. Subsequently, the plates were rinsed thrice and immersed in avidin-biotin-peroxidase complex (ABC) for 30 min in a thermostat. Following a rinse with PBS solution and avoiding light, the samples were submerged with 3,3’,5,5’-tetramethylbenzidine (TMB) chromogenic substrate for 30 min. When the standards wells showed a blue gradient, the reaction was stopped by adding 100 μl/well termination solution. The absorbance of each group was measured at 450 nm, and the concentration of secretory protein was calculated through a reference standard curve.

### Statistical analysis

SPSS 16.0 statistical software was utilized to perform statistical analysis. Data was presented as mean ± SD. Statistical difference was compared between two groups by using the independent sample t-test. The associated data were analyzed with a one-way analysis of variance (ANOVA) with Tukey’s post hoc test using SPSS 22. Threshold of statistical difference was set at p value < 0.05.

### Data availability statement

The datasets presented in this study can be found in online repositories. The names of the repository/repositories and accession number(s) can be found below: https://www.ncbi.nlm.nih.gov/geo/, GSE160633.

## RESULTS

### Identification of ARDEGs in IUA

**Figure 1:**
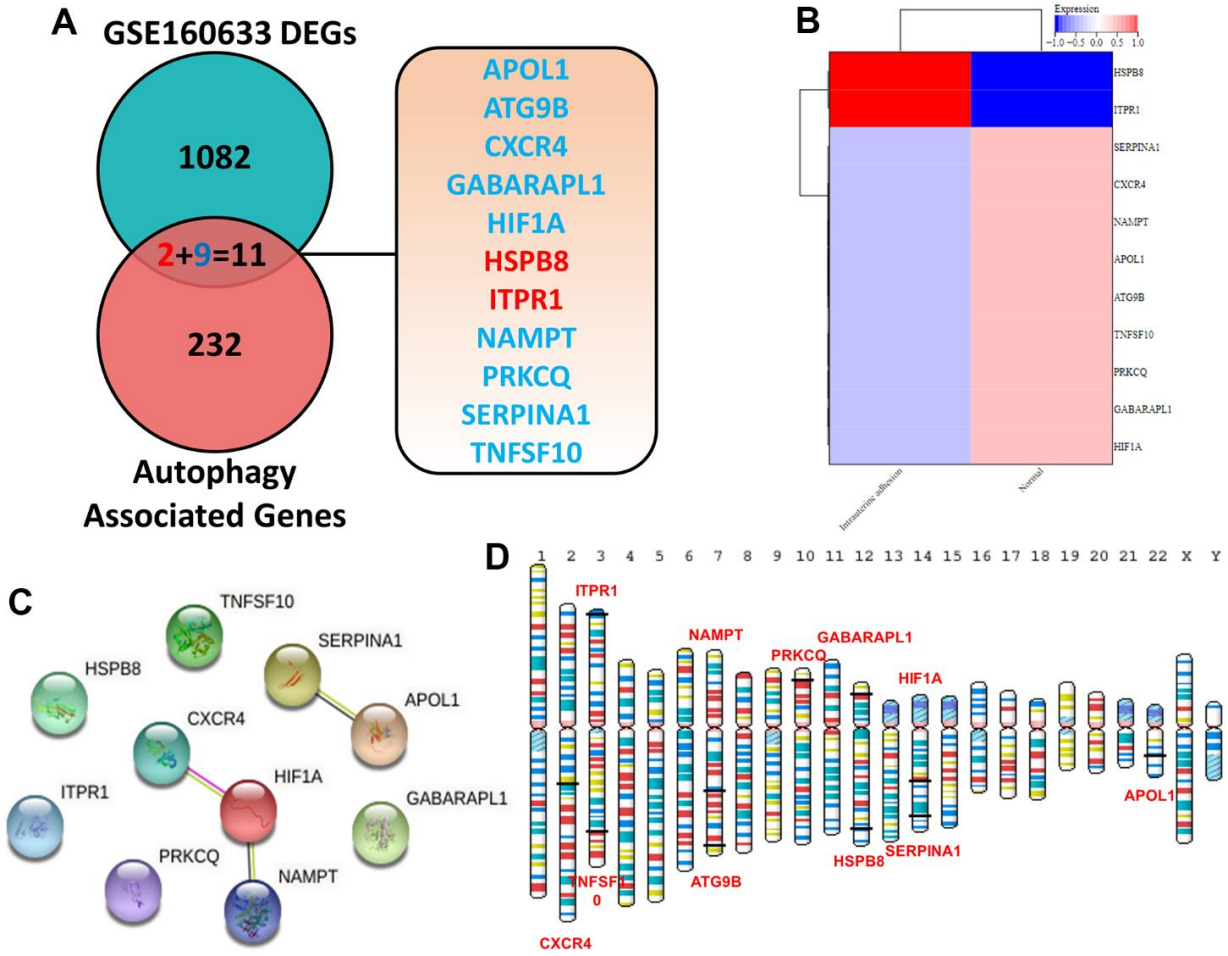
(**A**) Identification of 11 ARDEGs between DEGs and autophagy related genes. (**B**) Heatmaps of ARDEGs based on Log2FC. (**C**) Interaction between ARDEGs by PPI network (**D**) Location of ARDEGs on chromosomes.

As shown in Figure 1A, 1B, 1093 DEGs in IUA tissues was obtained from Supplementary Materials of previous study [18]. Furthermore, 11 overlapping genes were regarded as ARDEGs by Venn diagram analysis through taking the intersection between DEGs and 243 autophagy-related genes from Human Autophagy Database. Information of autophagy-related genes was shown in Supplementary Table 1. Heatmaps indicated that 11 ARDEGs contained 2 upregulated ARDEGs (HSPB8 and ITPR1) and 9 downregulated ARDEGs (SERPINA1, CXCR4, NAMPT, APOL1, ATG9B, TNFSF10, PRKCQ, GABARAPL1 and HIF1A).

### Autophagy-related PPI network construction and gene localization

To further understand the underlying interactions among 11 ARDEGs, STRING database was utilized to establish autophagy-related PPI network. After visualized by Cytoscape, 10 nodes (ARDEGs) and 3 edges (interactions) were included in autophagy-related network. This meant that there were regulatory relationships between them to affect the autophagy level in IUA. As illustrated in Figure 1D, the location of ARDEGs on the human chromosome was exhibited.

### Enrichment analysis of ARDEGs

**Figure 2:**
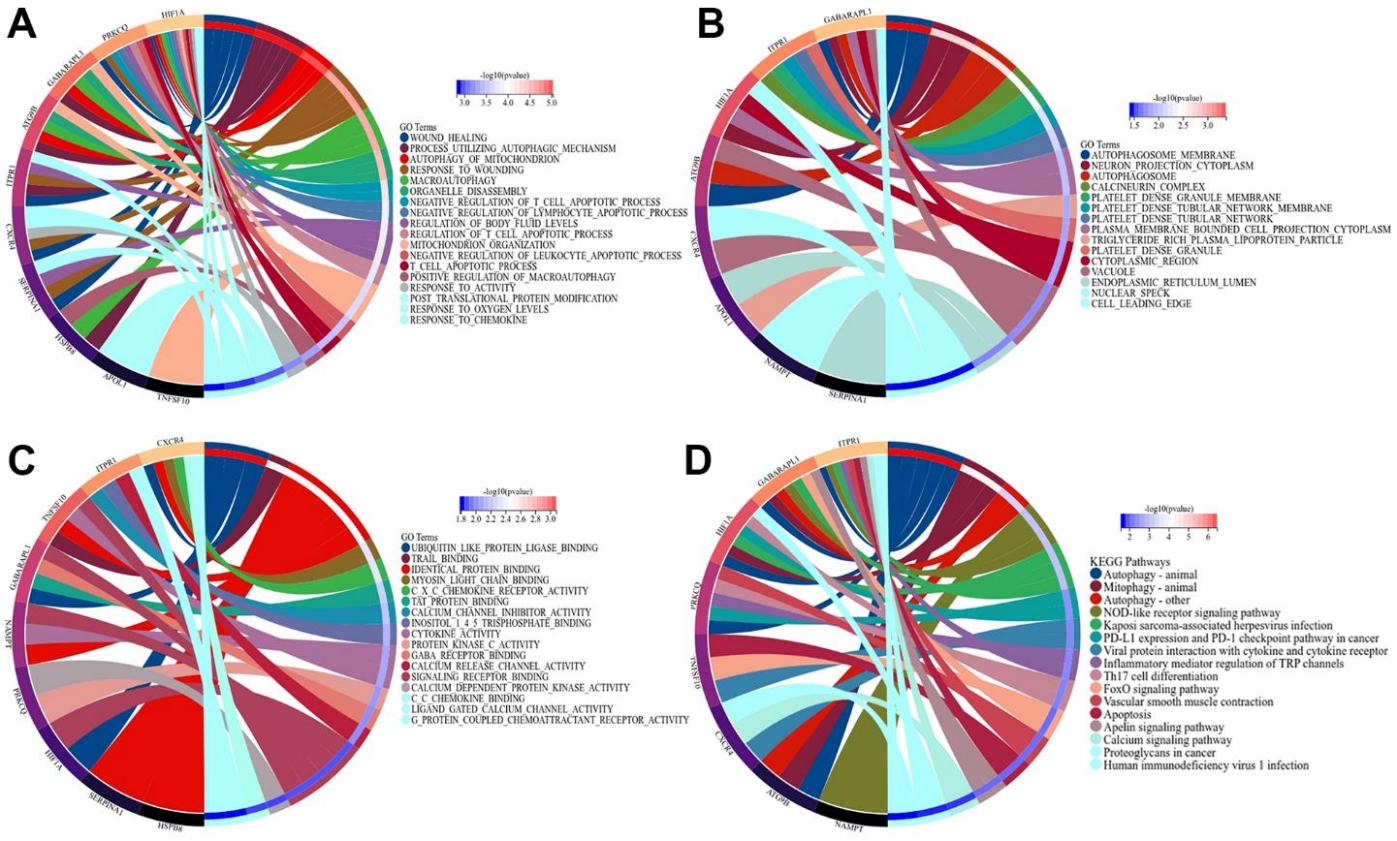
**GO and KEGG analysis of ARDEGs in IUA to reveal the function of ARDEGS.** (**A**) BP aspect; (**B**) CC aspect; (**C**) MF aspect; (**D**) KEGG analysis.

We performed GO and KEGG analysis of ARDEGs to further reveal their biological function. As demonstrated in Figure 2A–2D, for the BP-related terms, ARDEGs were primarily involved in response to chemokine, wound healing, mitochondrion organization; For CC-related terms, ARDEGs were mainly enriched in vacuole, autophagosome membrane, autophagosome; For the MF-related terms, DEGs were principally concerned with ubiquitin like protein ligase binding, identical protein binding, signaling receptor binding. Moreover, KEGG analysis verified that ARDEGs were involved in autophagy animal, mitophagy animal, nod-like receptor signaling pathway. GO analysis indicated that ARDEGs may regulate macrophage recruitment, uterine repair and mitophagy in IUA through autophagosomal formation and elongation of autophagosomal membrane. Mounting evidence indicates that actin dynamics and membrane-cytoskeleton scaffolds also have essential roles in macroautophagy. Branched actin polymerization is necessary for the biogenesis of autophagosomes from the endoplasmic reticulum (ER) membrane [25]. KEGG analysis also demonstrated that autophagy and mitophagy regulated by ARDEGs were involved in IUA. Our results were consistent with recent studies associated with autophagy in IUA and firstly bridged the potential relationship between mitophagy and IUA.

### Drug-ARDEGs network

**Figure 3:**
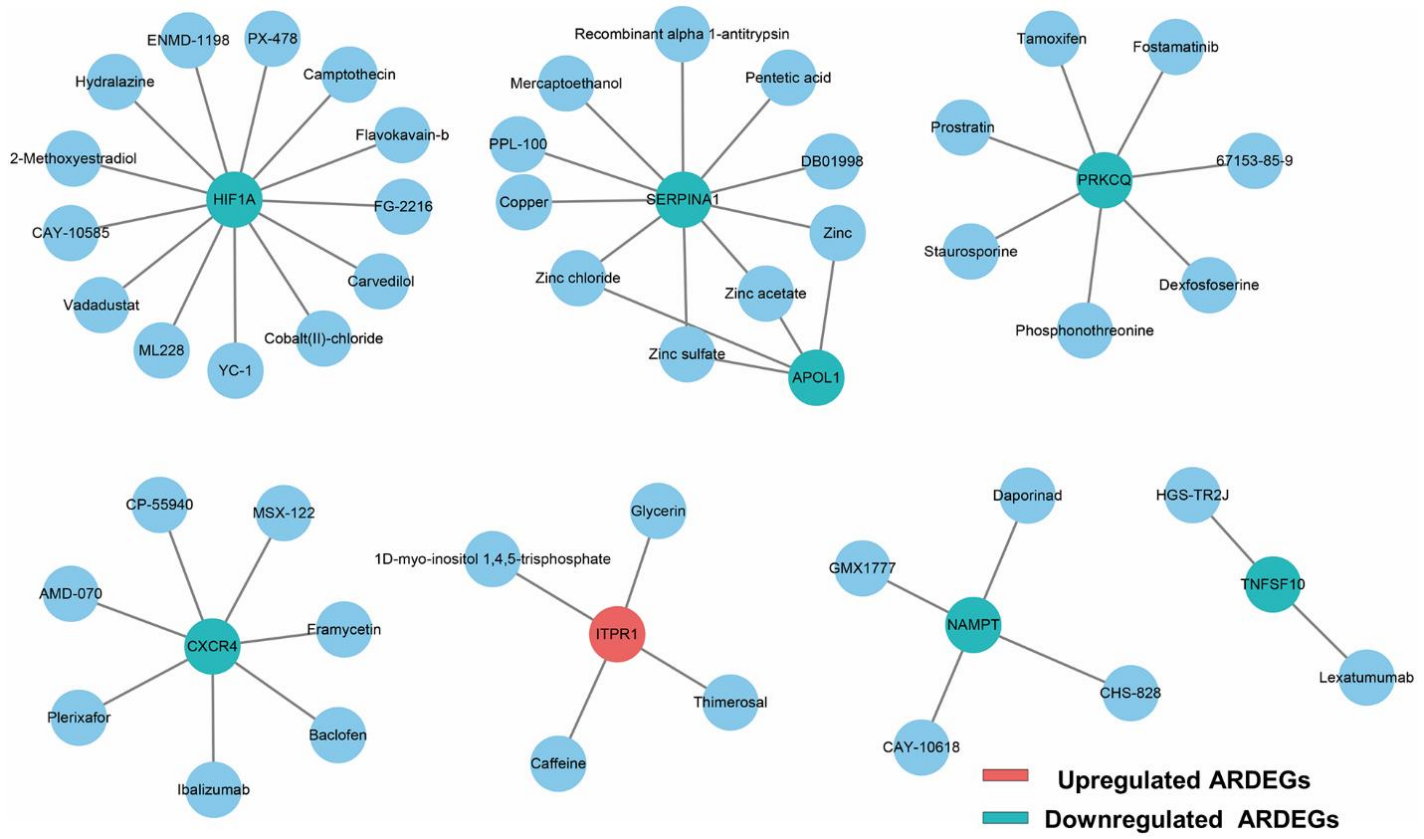
**Drug-ARDEGs network including 8 ARDEGs and 47 drugs.** Blue circles, potential target drugs; Red circles, upregulated ARDEGs; Blue-green circles, downregulated ARDEGs.

To explore potential drugs for regulating ARDEGs to achieve IUA target therapy, we obtained 47 predicted drugs from DGIdb database. For clarity of presentation, the network of the interaction between drugs and ARDEGs was established as exhibited in Figure 3. Unfortunately, the pharmacological mechanism and therapeutic effect of most above drugs should be further elucidated.

### Molecular docking

**Figure 4:**
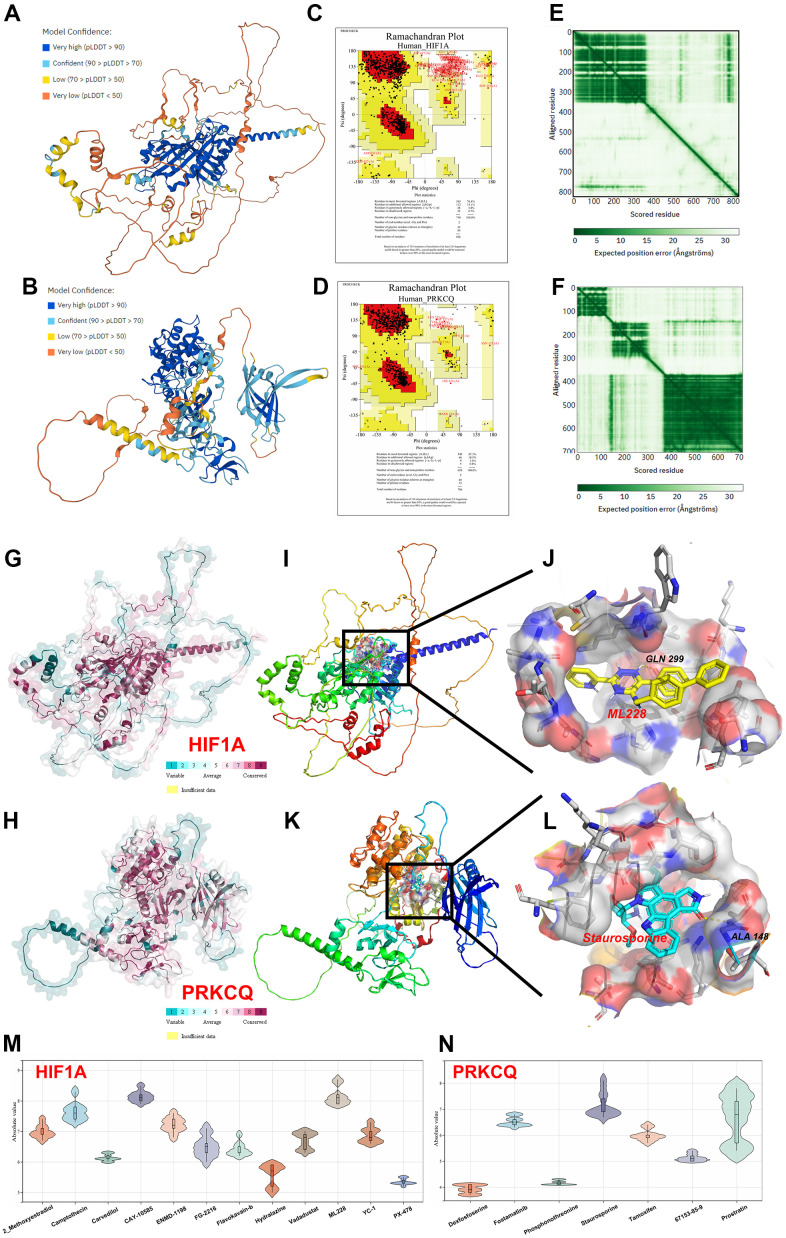
**Homology modeling and molecular docking.** The crystal structure and evaluation of (**A**) HIF1A and (**B**) PQKCQ. The ramachandran plot of (**C**) HIF1A structure. The ramachandran plot of (**D**) PQKCQ structure. The LDDT score of (**E**) HIF1A structure. The LDDT score of (**F**) PQKCQ structure. The assessment of protein conservation of HIF1A and PQKCQ was exhibited in (**G**, **H**) respectively. The best docking position between (**I**, **J**) ML228 and HIF1A or (**K**, **L**) staurosporine and PRKCQ were indicated. The absolute value of affinity between predicated small molecules and (**M**) HIF1A or (**N**) PQKCQ was shown.

With the progress of computer simulation, molecular docking pioneered a novel way to solve the problem that only very few drugs can be cultivated and identified for clinical treatment. As shown in Figure 4A–4H, Alphafold v2.0 predicted the crystal structure of human HIF1A and PRKCQ through AI technology. The results of LDDT score and ramachandran plot indicated that the predicted structures had great stereochemical quality. To screen the optimal drugs towards HIF1A and PRKCQ, molecular docking was performed by Autodock Vina software and we obtained affinity score to judge the relative strength of drugs-protein compounds. The high absolute affinity score suggested that the drug could bind more tightly to the protein. To achieve more direct visualization, the distribution of affinity scores of each drug was visualize violin plots as exhibited in Figure 4M, 4N. This allowed us to find that ML228 (7.9-8.7 kcal/mol, |interval score|)) and staurosporine (6.7-8.1 kcal/mol, |interval score|) had the highest binding affinity towards HIF1A and PRKCQ respectively. As demonstrated in Figure 4I–4L, the residues of HIF1A (GLN299) and PRKCQ (ALA148) were the druggable pockets to form hydrogen bonds and the perfect compounds. Furthermore, the conservation analysis of HIF1A and PRKCQ indicated that GLN299 and ALA148 had high conservation in multiply species. Above findings demonstrated that ML228 and staurosporine may be novel drugs for clinical treatment of IUA.

### Identification of ARDEMs in IUA

**Figure 5:**
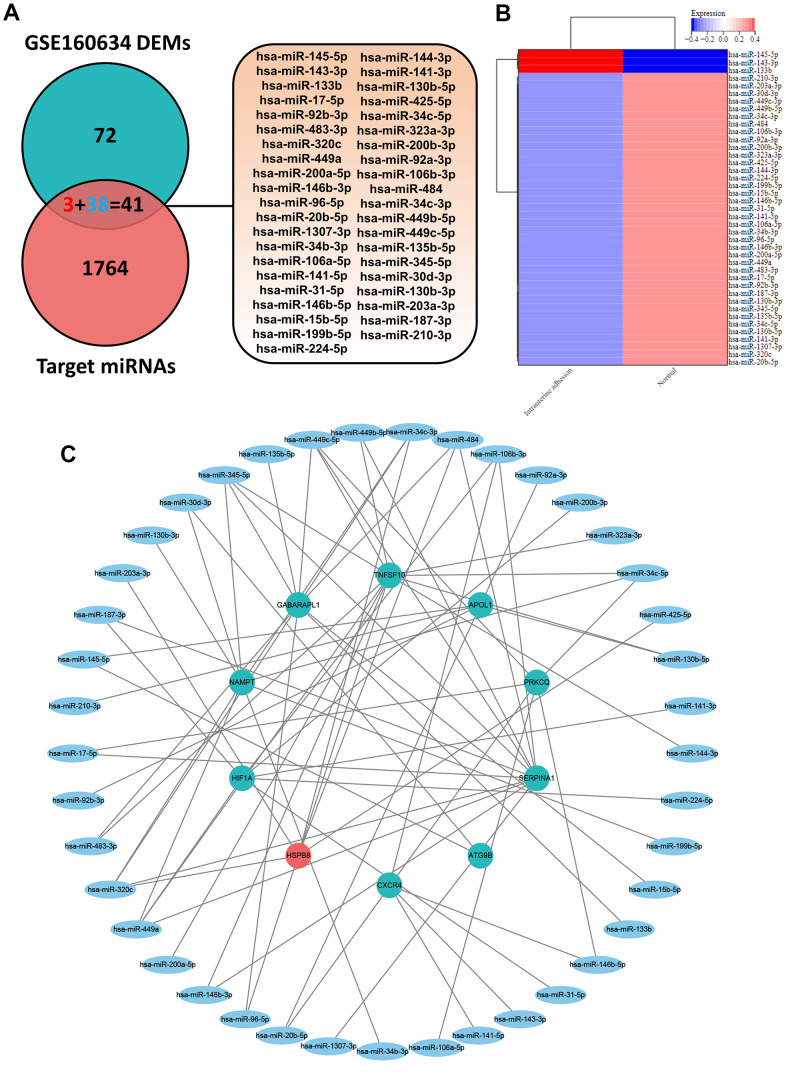
(**A**) Venn diagram of the intersection of DEMs and target miRNAs to obtain 41 ARDEMs. (**B**) Heatmaps of ARDEMs including 3 upregulated and 38 downregulated ARDEMs. (**C**) Construction of the autophagy related miRNA-mRNA regulatory network.

MiRNAs can regulate the post-transcriptional expression of target genes by binding to the 3ʹ-untranslated regions of messenger RNAs (mRNAs), leading to translational repression or mRNA cleavage [26]. Therefore, revealing the autophagy-related miRNA in IUA could help us deeply understand the underlying interaction between autophagy-related miRNAs and mRNAs. As shown in Figure 5A, 5B, 113 DEMs in IUA tissues was obtained from Supplementary Materials of previous study. Autophagy-related target miRNA was predicted based on 11 ARDEGs through miRWalk 3.0. Furthermore, 41 overlapping miRNAs were regarded as ARDEMs by Venn diagram analysis through taking the intersection between DEMs and 1805 autophagy-related target miRNA. Heatmaps indicated that 41 ARDEMs contained 3 upregulated ARDEMs and 38 downregulated ARDEMs.

### Autophagy-related miRNA–mRNA regulatory network establishment and hub ARDEMs analysis

**Figure 6:**
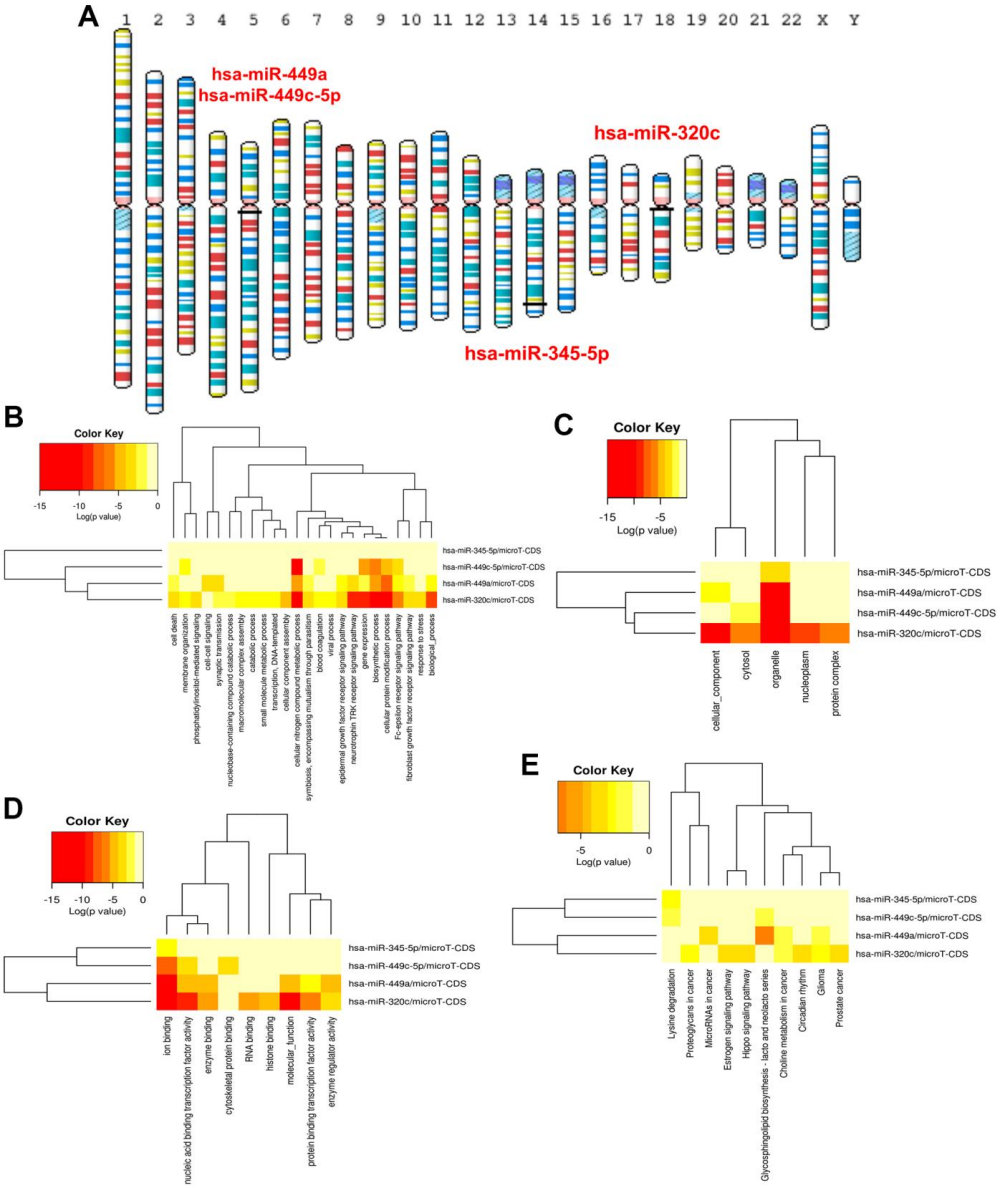
(**A**) Location of ARDEMs on chromosomes. GO and KEGG analysis of hsa-miR-320c, hsa-miR-449a, hsa-miR-449c-5p and hsa-miR-345-5p. (**B**) BP-terms, (**C**) CC-terms, (**D**) MF-terms and (**E**) KEGG pathways.

As shown in Figure 5C, the autophagy-related miRNA–mRNA regulatory network was established. In this network, upregulated and downregulated ARDEGs were represented by red and green circles, blue ellipses were on behalf of ARDEMs. 41 ARDEMs, 1 upregulated ARDEGs, and 9 downregulated ARDEGs were visualized. Among all ARDEMs, hsa-miR-320c, hsa-miR-449a, hsa-miR-449c-5p and hsa-miR-345-5p with the top number of regulated ARDEGs were regarded as hub ARDEMs. The location of hub ARDEMs on the human chromosome is indicated in Figure 6A. Enrichment analysis of hub ARDEMs demonstrated that BP-enriched terms included cellular nitrogen compound metabolic process, biosynthetic process and cellular protein modification process; CC-enriched terms were involved in organelle, cytosol, and cellular component; MF-enriched terms referred to ion binding and nucleic acid binding transcription factor activity. The results of KEGG analysis illustrated that hub ARDEMs participated in lysine degradation, glycosphingolipid biosynthesis-lacto and neolacto series, glioma and choline metabolism in cancer (Figure 6B–6E). Glycosphingolipid is a bioactive lipid that mediate key processes such as apoptosis and autophagy. Leng et al. reported that eliglustat could specifically block the synthesis of glycosphingolipid and autophagic degradation of TRAF3 to reduce osteoclastogenesis [27]. Furthermore, other metabolic process including the synthesis of cellular nitrogen compound, lysine [28] and choline [29] were also associated with autophagy [30]. Above results suggested that ARDEMs may affect autophagy through regulating various metabolic process.

### Hub circRNA selection and circRNA-miRNA-mRNA regulatory network construction

**Figure 7:**
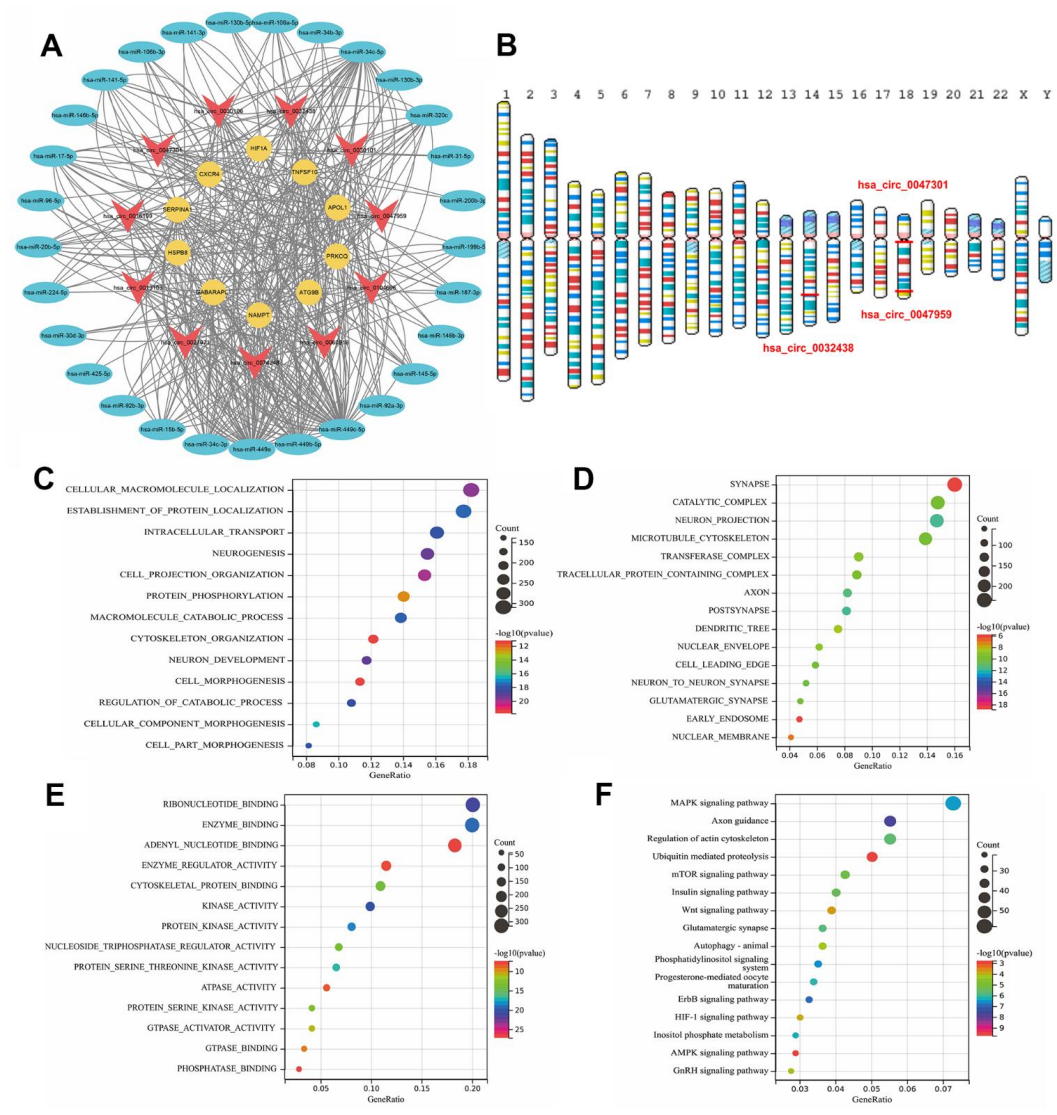
(**A**) Construction of the autophagy related circRNA-miRNA-mRNA regulatory network. (**B**) Location of ARDECs on chromosomes. GO and KEGG analysis of hsa-circ-0047959, hsa-circ-0032438, hsa-circ-0047301. (**C**) BP-terms, (**D**) CC-terms, (**E**) MF-terms and (**F**) KEGG pathways.

The circbank database was used to predict the corresponding circRNAs of miRNAs separately. After the intersection of the Venn diagram, intersection circRNAs were obtained. In addition, circRNAs with more than 6 regulated ARDEMs were identified as ARCs and included in the construction of autophagy-related circRNA-miRNA-mRNA regulatory network (Figure 7A). This network included 11 ARCs, 29 ARDEMs and 10 ARDEGs. Among the ARCs in this network, hsa-circ-0047959, hsa-circ-0032438, hsa-circ-0047301 regulated 10, 9 and 9 miRNAs respectively, and they were identified as hub ARCs. The location of hub circRNAs on the chromosome is shown in Figure 7B. KEGG and GO enrichment analysis showed that BP included cellular macromolecule localization, establishment of protein localization, intracellular transport and neurogenesis. CC included synapse, catalytic complex and neuron projection and microtubule cytoskeleton. MF included ribonucleotide binding, enzyme binding and adenyl nucleotide binding. KEGG included MAPK signaling pathway, axon guidance, regulation of actin cytoskeleton and ubiquitin mediated proteolysis. MAPK pathway was considered to be among the key mechanisms involved in autophagy [31]. Synaptic components were mediated by autophagy via modulation of their trafficking could contribute to wound healing [32]. Therefore, we believe that hub ARCs may MAPK pathway, neuromodulation and posttranslational modification to regulate the process of IUA via autophagy.

### Validation of hub circRNAs and hub DEMs with RT-PCR

**Figure 8:**
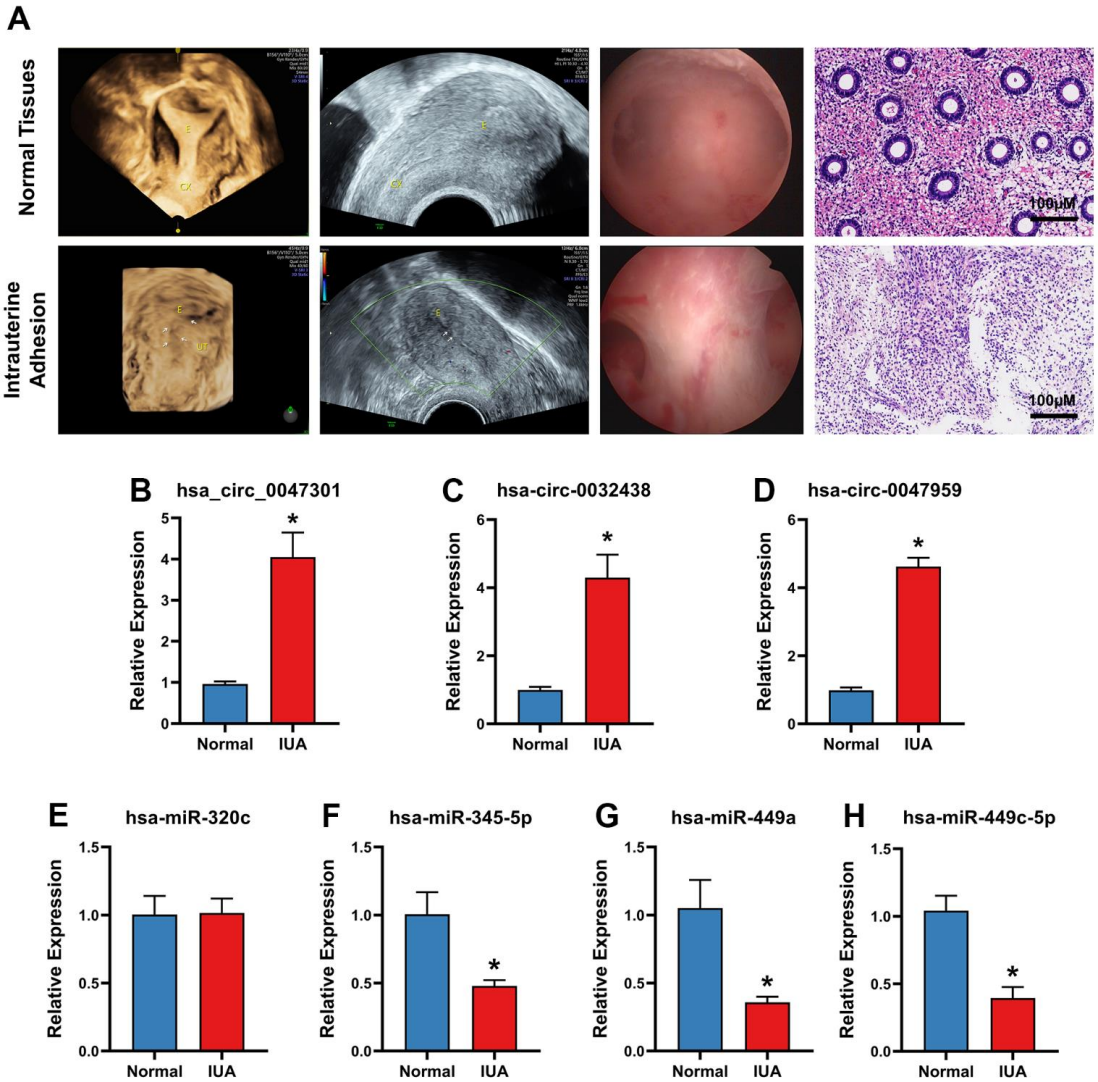
(**A**) B-ultrasound examination, hysteroscopy, Hematoxylin-Eosin Staining of intrauterine adhesion and normal uterine cavity. Validation of 3 ARDECs and 4 ARDEGs expression of RT-PCR. Relative expression level of (**B**) hsa-circ-0047301, (**C**) hsa-circ-0032438, (**D**) hsacirc-0047959 were obviously upregulated. Relative expression level of (**E**) hsa-miR-320c showed no significant statistical differences. Relative expression level of (**F**) hsa-miR-345-5p, (**G**) hsa-miR-449a and (**H**) hsa-miR-449c-5p were downregulated in IUA tissue. Data are presented as mean ± SD. *P < 0.05 compared to the normal tissues.

To validate hub ARCs and ARDEMs expression, RT-PCR was carried out in IUA tissues (n=5) and normal endometrium (n=5). As shown in Figure 8, all patients underwent B-ultrasound examination before surgery and the diagnoses of IUA patients were confirmed by hysteroscopy and pathomorphological examination through hematoxylin-eosin staining. As shown in Figure 8B, all three hub ARCs were markedly upregulated in IUA tissues when compared to normal endometrial tissue. Moreover, 3 hub ARDEMs (hsa-miR-345-5p, hsa-miR-449a and hsa-miR-449c-5p) were downregulated with the significant statistical differences. However, hsa-miR-320c had no significant statistical differences.

### The effect of hub ARCs on cell autophagy, myofibroblast transformation and collagen deposition

**Figure 9:**
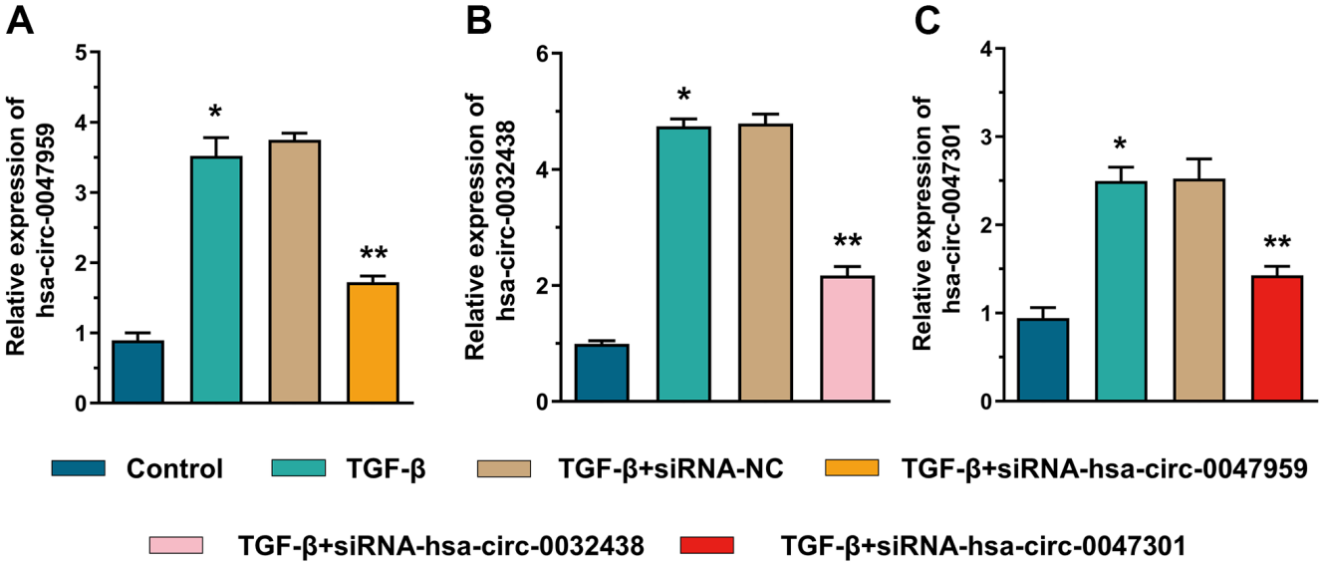
The siRNA interference efficiency of hsa-circ-0047959 (**A**), hsa-circ-0032438 (**B**), hsa-circ-0047301 (**C**).

**Figure 10:**
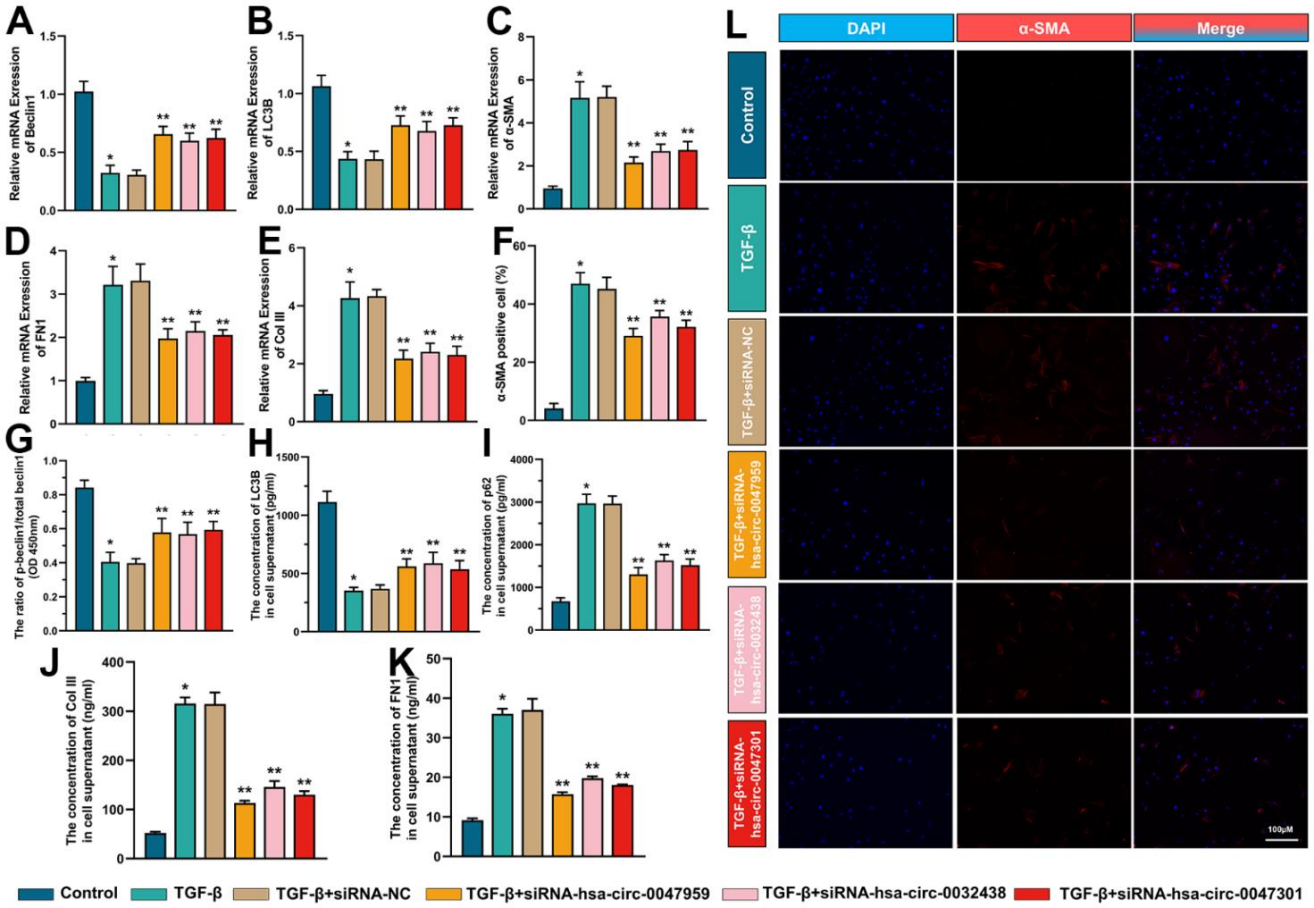
**The effect of hub ARCs on cell autophagy, myofibroblast transformation and collagen deposition.** The mRNA expression of Beclin1 (**A**) LC3B (**B**) α-SMA (**C**) FN1 (**D**) Col III (**E**) was detected through RT-PCR. The percentage of α-SMA positive cell was calculated in immunofluorescence results (**F**). The phosphorylation level of Beclin1 (**G**) protein expression of LC3B (**H**) and p62 (**I**) and release of Col III (**J**) and FN1 (**K**) was evaluated through ELISA. The immunofluorescence images of α-SMA in ESCs (**L**). *p <0.05, compared with Control; **p <0.05, compared with TGF-β+siRNA-NC.

The interference efficiency was indicated in Figure 9. As shown in Figure 10, the ESCs were treated with TGF-β to mimics the intrauterine adhesion microenvironment to verify the function of hub ARCs through siRNA interference [33]. The interference efficiency was indicated in Figure 9. RT-PCR results demonstrated that the autophagy biomarkers (Beclin1 and LC3B) expression was decreased with the TGF-β treatment. Recent studies indicated that autophagy could effectively inhibit the development of intrauterine adhesion. After knockdown of hub ARCs in ESCs, the level of autophagy was significantly enhanced as shown in Figure 10. Consistent with our hypothesis, myofibroblast transformation (the percentage of α-SMA positive cell and expression of α-SMA mRNA) and collagen deposition (the mRNA expression and release of Col III and FN1) were increased in TGF-β stimulation and markedly relieved with the silencing of hub ARCs. We also found that the phosphorylation level of Beclin1 and the concentration of LC3B was decreased and the concentration of P62 in ESCs was increased after TGF-β treatment. After knockdown of 3 hub ARCs, the above results were reversed.” has added into.

## DISCUSSION

IUA is irreversible damage to the basal layer of the endometrium caused by invasive operations or infections in the uterine cavity, leading to partial or complete occlusion of the uterine cavity [34]. IUA is a common intrauterine disease in women of childbearing age, which can cause changes in menstrual conditions such as reduced menstrual flow and amenorrhoea, and lead to increased risk of infertility, spontaneous abortion, implantation failure after assisted reproduction, recurrent miscarriage and placental abnormalities, seriously affecting women’s physical and mental health [35]. Currently, the main treatment for IUA is surgery, but the vicious cycle of adhesion-separation-readhesion tends to develop after surgery [36]. The main post-operative measures to prevent re-adhesion include the placement of intrauterine devices (IUDs) to act as a physical barrier to isolation and the use of drugs such as oestrogen to promote endometrial growth. However, due to the lack of knowledge of the internal mechanisms of IUA progress, the existing treatment strategies have not yet been well suited to improve the prognosis of patients [37]. Therefore, there is an urgent need to further improve the understanding of the occurrence of IUA and screen pivotal non-coding RNAs and mRNAs associated with IUA. Exploring undiscovered ncRNAs can be translated into clinical use for early screening and targeted treatment of patients with IUA to improve their prognosis. Autophagy is the process by which proteins or organelles in the cytoplasm are transported to lysosomes for degradation [38]. Currently, studies found the close association between IUA and autophagy, but no autophagy-related circRNA-miRNA-mRNA regulatory network has been established. For the first time, we revealed the interaction between autophagy-related circRNAs, miRNAs and mRNAs in IUA, providing a theoretical basis for further research into the pathogenesis of IUA and revisiting the treatment of IUA from a new perspective, offering new ideas for more optimal treatment choices to better protect patients’ fertility.

11 ARDEGs (APOL1, ATG9B, CXCR4, GABARAPL1, HIF1A, HSPB8, ITPR1, NAMPT, PRKCQ, SERPINA1, TNFSF10) were screened by overlapping the mRNA dataset and autophagy-related genes. Among these 11 DEGs, there were 2 up- regulated genes (HSPB8, ITPR1) and 9 down-regulated genes (APOL1, ATG9B, CXCR4, GABARAPL1, HIF1A, NAMPT, PRKCQ, SERPINA1, TNFSF10).

ATG9B is anchored to the cell membrane and is involved in the formation and transport of autophagosomes. It has been shown that bleomycin induces an increase in autophagic activity and upregulation of autophagy-related gene ATG9B expression during pulmonary fibrosis, which is involved in the development of pulmonary fibrosis [39]. In addition, down-regulation of HPV 16E6/E7 (16E6/E7) can inhibit the expression of autophagy gene ATG9B, thereby inhibiting cell proliferation and promoting early cervical cancer cell apoptosis [40]. In gastric and colorectal cancers, ATG9B has frameshift mutations, suggesting that these mutations may affect cancer progression by regulating autophagy [41]. As an inflammatory cytokine, studies reported that CXCR4 was closely related to physiopathological processes such as embryonic development, angiogenesis and inflammatory response, and has the ability to promote cell migration and adhesion, angiogenesis and endothelial proliferation [42]. Some studies have reported that CXCR4 plays an important role in regulating the immune inflammatory response in the fetal-maternal microenvironment [43]. Previous studies found that NAMPT is produced by lipid cells in the visceral lipid tissue, but also by the fetal membrane, amniotic epithelium, placenta and myometrium [44]. NAMPT and its pathway-related factors are widely involved in the inflammatory regulatory response of the mother and fetus during pregnancy.

Enrichment analysis indicated that ARDEGs were closely related to chemokine, oxygen levels, wound healing, process utilizing autophagic mechanism and response to wounding, which were closely related to the pathogenesis of IUAs, including endometrial damage and endometrial inflammation. The results also demonstrated that ARDEGs were enriched in cell leading edge and neuron projection cytoplasm, which were related to neuroreflex theory in the pathogenesis of IUAs. The neuroreflex theory was first proposed by Dr. Asherman in 1948, who suggested that the operation of the uterine cavity could lead to tonic contractions of the smooth muscles of the uterus through neuroreflexes, and that persistent tonic contractions of the uterine muscles could transform the uterine cavity from a physiological stenosis to an organic stenosis, leading to symptoms such as amenorrhoea and hypomenorrhoea [45]. This theory is the earliest of the many theories on the pathogenesis of IUAs, but there were few experimental studies to support it with valid experimental data.

While many drugs are used to treat IUAs, such as hormones, they are symptomatic drugs due to lack of well-defined target and always bring side-effect as follows: vaginal bleeding, breast cancer and thrombosis [46]. Therefore, there is an urgent need to discover target drugs for IUA. Based on ARDEGs, the DGIdb database was used to identify 47 FDA-approved drugs. Given the top 2 ranking of target drugs for ARDEGs, HIF1A and PRKCQ, molecular docking was applied to screen the best candidates. In this study, ML228 and staurosporine were found to have the highest binding affinity towards HIF1A and PRKCQ, respectively. ML228 is a potent activator of the HIF signalling pathway [47], activating both HIF and downstream EGFR [48], a transmembrane receptor that binds to epidermal growth factor to form a dimer that promotes mitosis, cell proliferation and participates in a variety of signalling pathways related to healing. Studies have shown that EGFR can promote the repair of gastric mucosa damage, maintain the integrity of the epithelial tissue of the gastrointestinal tract, and inhibit gastric acid secretion [49]. It also plays a role in mucosal repair of colonic inflammation in patients with ulcerative colitis [50]. It is hypothesized that it may play a role in the repair of endometrial damage, reducing the occurrence of IUAs. Additionally, staurosporine is an ATP competitive kinase inhibitor and can induce cell apoptosis in multiple cancer [51], which suggest it may contribute to myofibroblast apoptosis and decrease collagen deposition. The molecular docking results might provide medical relevance for treatment of IUA. We expected that the above results could provide more insights and therapeutic targets.

Accumulating evidence has indicated that dysregulation of miRNAs is involved in IUA. For example, miR-1291 promoted endometrial fibrosis by regulating the ArhGAP29-RhoA/ROCK1 signaling pathway in a murine model [16]. 113 DEMs were screened by the miRNA microarray for IUA, and 1764 autophagy-related miRNAs were predicted from 11 ARDEGs. After overlapping them, 41 ARDEMs were screened out. And then, autophagy-related miRNA-mRNA network was constructed. The regulatory network included 41 ARDEMs, 1 upregulated ARDEGs, and 9 downregulated ARDEGs. Although most of the identified mRNAs as well as miRNAs are downregulated, the reasons for this phenomenon may be attributed to NamiRNAs, which is a kind of miRNAs and their function is an enhancer trigger by modifying chromatin status that are favorable for transcriptional gene activation [52]. Therefore, when the inhibition of these miRNA by circRNAs, the expression of mRNA will also be downregulated [53]. In this network, hsa-miR-320c, hsa-miR-449a, hsa-miR-449c-5p and hsa-miR-345-5p with the most regulated target genes were identified as hub ARDEMs. hsa-miR-320c regulated SERPINA1, GABARAPL1, NAMPT and HSPB8. hsa-miR-449a regulated SERPINA1, TNFSF10, HIF1A, NAMPT. hsa-miR-449c-5p regulated SERPINA1, TNFSF10, PRKCQ, GABARAPL1. hsa-miR-345-5p regulated SERPINA1, TNFSF10, GABARAPL1, NAMPT. Enrichment analysis of hub miRNAs included gene expression, biosynthetic process and cellular protein modification process, ion binding and nucleic acid binding transcription factor activity. As the hub ARDEMs, hsa-miR-345-5p can mediate inflammatory response and participate in the occurrence and development of fibrotic diseases. miR-345-3p can alleviate the inflammatory response in endothelial cells with the involvement of the NF-κB pathway in atherosclerosis [54]. It also acts as an anti-inflammatory regulator in experimental allergic rhinitis via the TLR4/NF-κB pathway [55]. In fibrotic diseases, miR-345-5p mediates the activation of hepatic stellate cells and fibrotic livers by targeting HIF-1α, which subsequently modulates TGFβ/Smad2/Smad3 signaling [56].

Over the last few decades, functions of circRNAs became one of the most discussed issues and were considered as a novel biomarker of multiple diseases [57]. Accumulated evidence indicated that circRNAs functioned as a sponge of miRNA to involve in post-transcriptional regulation in IUA. Recent study has already investigated on the circular RNA profiles of IUA. Xie et al. indicated that circPlekha7 played an anti-fibrotic role in IUA [15]. Zheng et al. revealed that circPTP4A2-miR-330-5p-PDK2 signaling improved the repair of damaged endometrium [14]. In our study, to investigate the autophagy-related circRNAs and reveal their regulatory mechanism, autophagy-related circRNA-miRNA-mRNA network was established. The complicated network provided us a novel insight to explain the competing endogenous RNA interplay between ARCs and ARDEMs.

3 up-regulated circRNAs (hsa-circ-0047959, hsa-circ-0032438, hsa-circ-0047301) were identified as hub ARCs, according to the number of regulated miRNAs. Functional annotation analysis of hub ARCs indicated the BP enrichment from cellular macromolecule localization, establishment of protein localization, intracellular transport and neurogenesis. CC included synapse, catalytic complex and neuron projection and microtubule cytoskeleton. MF included ribonucleotide binding, enzyme binding and adenyl nucleotide binding. KEGG included MAPK signaling pathway, axon guidance, regulation of actin cytoskeleton and ubiquitin mediated proteolysis. As there are few studies on hub ARCs, we mainly analyzed their downstream miRNAs to further reveal the possible functions of hub ARCs.

Hsa-circ-0047301 regulated hsa-miR-320c. Previous study revealed that hsa-miR-320c was significantly down-regulated in keloid fibroblasts and contributed to collagen deposition [58]. As the upstream of hsa-miR-320c, hsa-circ-0047301 may sponge to hsa-miR-320c and achieve miRNA silence. Therefore, downregulation of hsa-circ-0047301 could be a novel perspective to reduce collagen deposition and IUA.

Hsa-circ-0032438 regulated hsa-miR-449c-5p and hsa-miR-449a, both of them are hub ARDEMs. Hsa-miR-449c-5p is rarely investigated, and limited studies suggest that it is associated with osteogenic differentiation of valve interstitial cells [59] and the progression of hepatocellular carcinoma [60]. Many studies pointed out that miR-449c-5p was involved in inflammatory response. Fu et al. revealed that miR-449c-5p could alleviate HUVECs injury through inhibiting the NF-κB signaling pathway activation by targeting TAK1 [61]. Moreover, previous study also proved that miR-449c-5p participated in the activation of microglia and inflammatory response to cerebral ischemia [62]. miR-449a, located on chromosome 5q11, has been reported to be dysregulated in many diseases and adjust cell apoptosis, proliferation, autophagy and inflammation by influencing downstream genes expression [63]. miR-449a could induce inflammatory factors release in the spinal cord of spinal cord injury rats [64]. Also, miR-449a played a crucial role in the progression of pulmonary fibrogenesis [63]. The characteristic of IUA is endometrial fibrosis. Excessive inflammatory reaction aggravated IUAs. Therefore, it can be speculated that hsa-circ-0032438/has-miR-449a pathway may be the key signaling for the treatment of IUA. Above results of microarray analysis were in accordance with previous studies and provided supportive evidence for further analysis.

RT-PCR was performed to validate expression level of hub ARCs and ARDEMs in IUA. In comparison of normal endometrial tissue, all three hub ARCs were upregulated in IUA tissue. Except, all hub ARDEMs were downregulated. No significant statistical difference was found in expression has-miR-320c.

*In vitro* experiments were investigated to explore the effect of hub ARCs on cell autophagy, myofibroblast transformation and collagen deposition. The results demonstrated that the decrease of autophagy and increase of α-SMA, Col III and FN1 with the treatment TGF-β to mimics IUA microenvironment. siRNA silence improved the mRNA expression of beclin1 and LC3B and decreased myofibroblast transformation and collagen deposition. These results suggested that interfering hub ARCs could restore autophagy activity, inhibit the differentiation of ESCs into myofibroblasts and reduce the formation of intrauterine adhesion. Therefore, the potential function of hsa-circ-0047959, hsa-circ-0032438, hsa-circ-0047301 is worth to be explored and regards as the novel target of clinical treatment.

In our study, some limitations were inevitable. the therapeutic effect of potential drugs was only performed simulation prediction rather than experimental verification. For LC3B detection, the change of LC3B II protein expression could be detected more precisely by western blot. To clarify the co-expression and interaction between ARDEMs and ARDEGs in IUA, luciferase assay should be performed in further study. Furthermore, the diagnostic efficiency of hub ARCs and ARDEMs was not clinically validated for there was no worldwide IUA clinical database. Therefore, the link between the circRNAs and predicted miRNAs or mRNAs in IUA should be deeply explored in further study through rigorous experimental confirmations *in vitro* and *vivo*.

## CONCLUSIONS

In current study, we firstly constructed autophagy-related circRNA-miRNA-mRNA regulatory network and identified hub ARCs and ARDEMs that have not been reported in IUA. We hope our study can provide novel biomarkers to reveal the underlying mechanism and therapeutic strategy for IUA.

## Supplementary Material

Supplementary Figure 1

Supplementary Table 1

Supplementary Table 2
