# Supplementary material for: Integrative analysis links autophagy to intrauterine adhesion and establishes autophagy-related circRNA-miRNA-mRNA regulatory network
Source: Aging (Albany NY). 2023 Aug 23;15(16):8275–97. doi: 10.18632/aging.204969 (PMC10497020; doi:10.18632/aging.204969)
Supplement: Supplementary Figure 1 [file aging-15-204969-s001.pdf]

SUPPLEMENTARY FIGURE

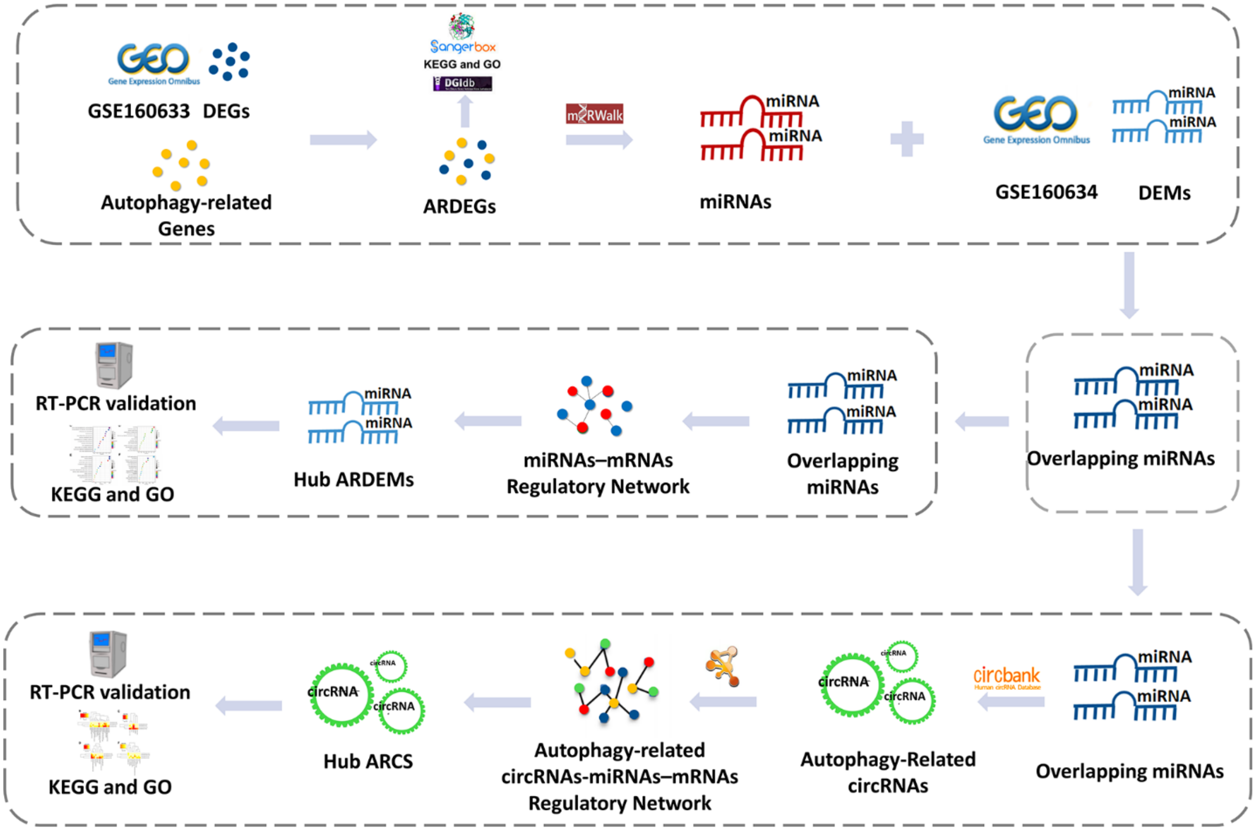

Supplementary Figure 1. The schematic workflow of Autophagy-related Functional circRNA—miRNA—mRNA network in intrauterine adhesion.
