# Supplementary material for: Integrative analysis links autophagy to intrauterine adhesion and establishes autophagy-related circRNA-miRNA-mRNA regulatory network
Source: Aging (Albany NY). 2023 Aug 23;15(16):8275–97. doi: 10.18632/aging.204969 (PMC10497020; doi:10.18632/aging.204969)
Supplement: Supplementary Table 2 [file aging-15-204969-s003.pdf]

Supplementary Table 2. The primer sequences of hub circRNAs and hub DEMs.

hsa-miR-320c	
FORWARD	GCACGTCCAAAAGCTGGGTTG
REVERSE	ATCCAGTGCAGGGTCCGAGG
hsa-miR-449c-5p	
FORWARD	AAGCGGATAGGCAGTGTATTGCT
REVERSE	ATCCAGTGCAGGGTCCGAGG
hsa-miR-449a	
FORWARD	AGCTGGACTGGCAGTGTATTGTT
REVERSE	ATCCAGTGCAGGGTCCGAGG
hsa-miR-345-5p	
FORWARD	AAGTTGCAGCTGACTCCTAGTCC
REVERSE	ATCCAGTGCAGGGTCCGAGG
hsa-circ-0047301	
FORWARD	AGGCTGTTCAAACACAAGCG
REVERSE	ACGCAGCATCACATTCACTG
hsa_circ_0032438	
FORWARD	TCAGAGACCATGCTGTTGGATC
REVERSE	AAGGCTGAGCAAAAGCATGG
hsa_circ_0047959	
FORWARD	TTTGCAACAGCTCAGCTTGG
REVERSE	ACAAAAGCAACCCCACAGAC
